# Anti-virulence potential of iclaprim, a novel folic acid synthesis inhibitor, against *Staphylococcus aureus*

**DOI:** 10.1007/s00253-024-13268-2

**Published:** 2024-08-05

**Authors:** Lingyun Hao, Jingwen Zhou, Han yang, Chunyan He, Wen Shu, Haoyue Song, Qingzhong Liu

**Affiliations:** 1 Department of Clinical Laboratory, Shanghai General Hospital, Shanghai Jiaotong University School of Medicine, Shanghai, China; 2 Department of Clinical Laboratory, Shanghai Municipal Hospital of Traditional Chinese Medicine, Shanghai University of Traditional Chinese Medicine, 274 Zhijiang Middle Rd., Shanghai, 200071 China

**Keywords:** *Staphylococcus aureus*, Iclaprim, Sub-inhibitory concentration, Anti-virulence, Antibiofilm

## Abstract

**Abstract:**

Infections caused by *Staphylococcus aureus* pose a significant global public problem. Therefore, new antibiotics and therapeutic strategies are needed to combat this pathogen. This investigation delves into the effects of iclaprim, a newly discovered inhibitor of folic acid synthesis, on *S*. *aureus* virulence. The phenotypic and genotypic effects of iclaprim were thoroughly examined in relation to virulence factors, biofilm formation, and dispersal, as well as partial virulence-encoding genes associated with exoproteins, adherence, and regulation in *S*. *aureus* MW2, N315, and ATCC 25923. Then, the *in vivo* effectiveness of iclaprim on *S*. *aureus* pathogenicity was explored by a *Galleria mellonella* larvae infection model. The use of iclaprim at sub-inhibitory concentrations (sub-MICs) resulted in a reduction of α-hemolysin (Hla) production and a differential effect on the activity of coagulase in *S*. *aureus* strains. The results of biofilm formation and eradication assay showed that iclaprim was highly effective in depolymerizing the mature biofilm of *S*. *aureus* strains at concentrations of 1 MIC or greater, however, inhibited the biofilm-forming ability of only strains N315 and ATCC 25923 at sub-MICs. Interestingly, treatment of strains with sub-MICs of iclaprim resulted in significant stimulation or suppression of most virulence-encoding genes expression. Iclaprim did not affect the production of δ-hemolysin or staphylococcal protein A (SpA), nor did it impact the total activity of proteases, nucleases, and lipases. *In vivo* testing showed that sub-MICs of iclaprim significantly improves infected larvae survival. The present study offered valuable insights towards a better understating of the influence of iclaprim on different strains of *S. aureus*. The findings suggest that iclaprim may have potential as an anti-virulence and antibiofilm agent, thus potentially mitigating the pathogenicity of *S*. *aureus* and improving clinical outcomes associated with infections caused by this pathogen.

**Key points:**

• *Iclaprim effectively inhibits α-hemolysin production and biofilm formation in a strain-dependent manner and was an excellent depolymerizing agent of mature biofilm*

• *Iclaprim affected the mRNA expression of virulence-encoding genes associated with exoproteins, adherence, and regulation*

• *In vivo study in G. mellonella larvae challenged with S. aureus exhibited that iclaprim improves larvae survival*

**Supplementary Information:**

The online version contains supplementary material available at 10.1007/s00253-024-13268-2.

## Introduction

Antibiotic resistance caused by the widespread and irrational use of antimicrobial drugs, especially the emergence and prevalence of multi-drug resistant (MDR) bacteria, poses a great challenge for clinical anti-infection treatment and makes it emergency to develop novel antibiotic alternatives (Nicolas et al. 2019).

Iclaprim is a novel selective inhibitor of microbial dihydrofolate reductase (DHFR) known for synthesizing thymine used for folate biosynthesis and DNA replication (Noviello et al. 2018). Its efficacy against *Staphylococcus aureus* (including methicillin-resistant *S*. *aureus*, MRSA) has been demonstrated both *in vitro* and *in vivo* (Huang et al. 2020, 2018; Laue et al. 2009; Noviello et al. 2019). Additionally, iclaprim displays the potential to repress the production of toxin, such as α-hemolysin (Hla), of *S*. *aureus* (Bryant et al. 2019). Owing to these promising results, iclaprim has been undergoing Phase 3 clinical assessment for treating bacterial skin and soft infections as well as nosocomial pulmonary infection (Huang and Dryden 2018; Wang et al. 2022).

*S*. *aureus* is widely recognized for its formidable pathogenic properties attributed to the production of a diverse array of virulence factors, encompassing toxins (such as hemolysin), extracellular enzymes (such as protease, nuclease, and lipase), surface adhesion molecules (such as staphylococcal protein A, clumping factors, and fibronectin-binding proteins), and regulators (such as accessory gene regulator and staphylococcal accessory regulator A) (Cheung et al. 2021). The impact of antibiotics on bacterial virulence factor production during anti-infective therapy bears great significance for disease outcomes (Chen et al. 2021). As a result of drug pharmacokinetics, drug tissue penetration, drug-drug interaction, and the emergence of resistant pathogens, bacteria are frequently exposed to sub-inhibitory concentrations (sub-minimal inhibitory concentrations, sub-MICs) of drugs during antibiotic treatment (Chen et al. 2021). It is well-documented that sub-MICs of antimicrobial agents can exert pivotal effects on the expression of bacterial virulence factors (Chen et al. 2021). Despite the confirmed anti-bacterial activity and limited inhibition of toxin expression, the comprehensive effects of iclaprim on *S*. *aureus* virulence remain inadequately explored.

Henceforth, this study delved into the impact of iclaprim on the virulence of *S*. *aureus*. This includes an exploration of the expression of primary virulence genes, the production of toxins, surface protein and exoenzymes, the formation and eradication of biofilm, and the effects of *in vivo* anti-virulence.

## Materials and methods

### Strains and antimicrobial agent

*S. aureus* strains MW2 (ATCC BAA-1707), ATCC N315 and ATCC 25923 selected in this study were gifts from Professor Fangyou Yu, based at the Shanghai Pulmonary Hospital, School of Medicine, Tongji University. The strains are generally recognized as clinical strains for research purposes. Iclaprim was purchased from MedChemExpress (Monmouth Junction, NJ, USA), which was dissolved in dimethyl sulfoxide (DMSO) and diluted with sterile water to the experimental concentrations.

### Determination of minimal inhibitory concentrations

The minimal inhibitory concentrations (MICs) of iclaprim for experimental strains were detected in triplicate using the broth microdilution method in cation-adjusted Mueller–Hinton broth (Becton–Dickinson, Franklin Lakes, NJ, USA) in accordance with the Clinical and Laboratory Standards Institute (CLSI) protocols (Humphries et al. 2021). Based on a study by Huang et al. (2017), the susceptibility of iclaprim (MIC ≤ 1 μg/mL) was confirmed.

### Growth kinetic curves

*S*. *aureus* strains were cultured overnight in brain heart infusion broth (BHI, OXOID, Basingstoke, Hampshire, UK) at 37 °C with shaking at 200 rpm. The next day, the cultures were diluted 1:100 into 30 mL fresh BHI, followed by addition of different concentrations of iclaprim (1 MIC, 1/2 MIC, 1/4 MIC, 1/8 MIC, 1/16 MIC, and 1/32 MIC). The diluted cultures were incubated at 37 °C with shaking at 200 rpm. Bacteria growth was monitored by measuring the absorbance at 600 nm (OD_600_) at hourly intervals using a UV-2102C ultraviolet spectrophotometer (Unico Instruments, Shanghai, China). Student’s *t* test was used to analyze the difference of bacterial growth.

### Hemolysis activity detection

*S. aureus* strains were cultured in tryptic soy broth (TSB) medium (OXOID, Basingstoke, Hampshire, UK) supplemented with graded sub-MICs of iclaprim at 37 °C, 200 rpm. Once the cultures reached the desired growth phase, supernatants were collected and filtrated with 0.22 µm filter for the subsequent use. Then, the activity of α-hemolysin (Hla, using defibrinated rabbit blood, Shanghai Yuduo Biotechnology Co. LTD, Shanghai, China) and δ-hemolysin (Hld, using human erythrocytes, Shanghai Yuduo Biotechnology Co. LTD) was detected using previously established methods (Liu et al. 2018). Culture filtrate (50 µL) was mixed with blood red cells (50 µL) in a sterile 96-well plate (Corning, Shanghai, China) and incubated for 1 h at 37 °C. Following centrifugation (3500 rmp, 5 min), the supernatant was transferred to a new plate, and the absorbance value of OD_540_ was measured. Triton-X 100 (0.5%) and phosphate-buffered saline (PBS) were employed as positive and negative controls, respectively. All detections were performed in triplicate. The unmedicated culture filtrate served as the 100% hemolysis control, and the hemolysis rate (% hemolysis) was calculated against the control culture.

### Hla production detection

The levels of Hla were detected by means of a specific sandwich enzyme-linked immunosorbent assay (ELISA) (Jin et al. 2018) in strict consideration of the instructions of the kit (Shanghai Jianglai Biotechnology Co., Ltd, Shanghai, China). In brief, the filtrates or standards were added to the anti-Hla antibody coated microplate, followed by the peroxidase-conjugated anti-Hla polyclonal antibody, then incubated for 1 h at 37 °C. After 5 times of washing, 3,3′,5,5′-tetramethylbenzidine (TMB) substrate was added and colored for 15 min at 37 °C and then stopped by addition of a stop solution. The absorbance due to the color change was measured at 450 nm. The absorbance was proportional to the concentration of the Hla. Sample concentrations were calculated using the standard curve (ng/mL, Fig. S1a). The Hla standard is a recombinant protein expressed by *Escherichia coli* (provided with the test kit).

### Staphylococcal protein A production detection

Staphylococcal protein A (SpA) was prepared using a previously described method (Cheung and Fischetti 1988). Briefly, bacterial cells collected from the “hemolysis assay” were lysed using lysis buffer [0.05 M Tris (PH = 7.5), 30% raffinose, 0.145 M NaCl] containing DNase (10 μg, Sangon Biotech, Shanghai, China), lysostaphin (100 μg, Sangon Biotech), iodoacetamide (final concentration of 1 μg/mL, Sigma-Aldrich, St. Louis, MO, USA), and benzoyl sulfonyl fluoride (PFSF, final concentration of 1 mM/mL, Sigma-Aldrich). The lysis mixture was incubated with 24 rmp at 37 °C for 1 h and then centrifuged at 8000 × g for 20 min. The supernatant was harvested for the detection of SpA by sandwich ELISA (Shanghai Jianglai Biotechnology Co., Ltd) according to the manufacture’s protocol. The SpA level was calculated refer to the standard curve (U/L, Fig. S1b). The recombinant SpA protein standards were supplied with the kit.

### Coagulase titer detection

Bacterial culture filtrates were harvested following the protocol described in “hemolysis assay.” The supernatant filtrates underwent serial dilution by twofold and were then combined with an equal volume (50 µL) of lyophilized rabbit plasma solution (Shanghai Yuduo Biotechnology Co. LTD) in a sterile 96-well plate, according to previous described methods (Liu et al. 2018). The presence of coagulase (Coa) was confirmed by observing the clots of the filtrate after 4 h of incubation at 37 °C. The titer was defined as the highest culture filtrate dilution producing observable clotting.

### Nuclease activity detection

The detection of nuclease activity in culture filtrates was accomplished using a toluidine blue DNase agar plate (Qingdao Haibo Biological Co., LTD, Qingdao, Shandong province, China) as described previously (Liu et al. 2018). To create wells of 5 mm in diameter, holes were dug into the plate. The culture filtrates were then added to the wells and incubated at 37 °C for 24 h. The activity of the nuclease was assessed based on the diameter (mm) of the baby pink circle surrounding the well.

### Protease activity detection

The total activity of protease was detected by the methods described by Liu et al. (2018). A 150 µL of supernatant filtrates was added to 1 mL chromogenic azocasein solution (1%, 0.05 M Tris–HCl, pH 7.5, Sigma-Aldrich), mixed, and incubated at 37 °C for 60 min. Then, the protein hydrolysis reaction was terminated by adding 500 µL of 10% (w/v) trichloroacetic acid (Sigma-Aldrich). After 30 min, the mixed solution was centrifuged at 10,000 × g for 10 min, and the OD_328_ of the supernatant was determined. The results were depicted as the percentage of proteolytic activity relative to that of the control (iclaprim-free).

### Lipase activity detection

A 50 µL of supernatant filtrate was added to 1.2 mL of 4-nitrobenzoic octanoate (Sigma-Aldrich) solution and incubated for 15 min at 37 °C; then, the absorbance value at 405 nm was determined (Liu et al. 2018). The lipase activity was described as a percentage of that of the drug-free control.

### Biofilm formation inhibition assay

#### Crystal violet staining

Detection of biofilm formation was performed using crystal violet (CV, Sangon Biotech) in a 96-well plate with minor modifications (Xu et al. 2016). The 0.5 McFarland concentration of the bacterial suspension was diluted 1 in 100 in TSB medium with 1% glucose (TSB-G) (Sigma-Aldrich). Iclaprim dilution was prepared in TSB-G at different sub-MICs. One hundred microliters of each of bacterial culture and the drug solution were added into 96-well plates. Each plate was set up with blank control (only medium) and positive control (bacteria + iclaprim-free medium), and incubated for 48 h at 37 °C. The culture fluid was gently aspirated, and each well was washed three times with sterile saline and then stained with 0.5% CV solution for 15 min at room temperature. The plate was washed again for removing the unincorporated dye and dried naturally. The incorporated CV of each well was extracted with 200 µL of 100% ethanol; then, the extracting solution was pipetted into a new plate, and the value of OD_595_ was measured.

#### Laser scanning confocal microscopy observation

The graded sub-MICs of iclaprim were added to each of confocal culture dishes (Becton–Dickinson); the controls were set as described in “crystal violet staining” subsection. The culture dishes were incubated for 48 h at 37 °C and washed three times. Then, the fluorescein isothiocyanate-conjugated concanavalin A (FITC-ConA) staining dye (100 μg/mL) (Sigma-Aldrich) was added into each dish and stained at 4 °C in the dark. After 30 min, the dishes were gently washed with sterile saline for removing the excess dye and dried at room temperature; then, 50 µL of anti-quenching agent was applied to each dish for preventing rapid fluorescence quenching. The formation of biofilm was evaluated by laser scanning confocal microscopy (LSCM) (Takenaka et al. 2001). All tests were carried out in triplicate.

### Biofilm eradication assay

The biofilm eradication of iclaprim was evaluated by LSCM, as described above. Briefly, the preparation of biofilm (without antibiotic) was carried out using confocal culture dishes, according to the methods under “LSCM observation.” After incubating for 48 h at 37 °C, the dishes were washed, and different concentrations of drug (1, 2, 5, 10, 15, 20, and 25 MICs) were added, followed by incubation for 24 h. After washing, fluorescent staining was performed, and the biofilm was observed using LSCM.

### Quantitative real-time polymerase chain reaction (qRT-PCR)

The effects of sub-MICs of iclaprim on the transcription of 18 representative virulence genes were evaluated. These factors included genes encoding Hla (*hla*), Coa (*coa*), SpA (*spa*), clumping factor A and B (*clfA* and *clfB*), fibronectin-binding proteins A and B (*fnbA* and *fnbB*), *S*. *aureus* surface protein G (*sasG*), serine-aspartate repeat family protein D (*sdrD*), autolysin A (*atlA*), intracellular adhesion A and D (*icaA* and *icaD*), *RNAIII*, accessory gene regulator A (*agrA*), *S*. *aureus* exoprotein expression S (*saeS*), staphylococcal accessory regulator A (*sarA*), sigma factor B (*sigB*), and repressor of toxin (*rot*). RNA extraction, cDNA synthesis, and qRT‑PCR were carried out using the primers listed in Table S1. All tests were examined in triplicate. Relative gene expression levels were analyzed by the 2^−ΔΔCT^ method described by Zhou et al. (2022).

### Galleria mellonella infection model

The *in vivo* protective role of iclaprim against *S*. *aureus* pathogenesis were assessed by the *Galleria mellonella* larvae infection model (Liu et al. 2018). To standardize the experiments, the larvae (Tianjin Huiyude Biotechnology Co., Ltd, Tianjin, China) weighting about 250–300 mg and without color alteration were selected. Single colonies of *S. aureus* strain MW2, N315, and ATCC25923 were respectively inoculated into BHI broth containing the 1/16 and 1/32 MICs of iclaprim and cultured at 37 °C with 220 rpm until an OD_600_ = 0.6. Cells were harvested, rinsed and resuspended to a bacterial inoculum with a density of 7.5 × 10^9^ CFU/mL. Larvae were injected with 10 μL of bacterial suspension treated with iclaprim using a microinjector at the third pair of hind prolegs. Controls larvae were challenged with10 μL of untreated bacterial inoculum (positive), or sterile PBS (negative). In each group, 10 larvae were used in 3 separate experiments. All larvae were incubated at 37 °C in the dark. Larval mortality was monitored daily for 6 consecutive days. Larvae were considered dead when they were unable to move, failure to respond to stimuli, and developed melanin deposits. Kaplan–Meier analysis was carried out to plot survival curves, and log-rank test was performed to compare the differences of the survival. A *P* value of less than 0.05 was considered as statistically significant.

## Results

### Effect of iclaprim on the growth of *S. aureus*

**Fig. 1:**
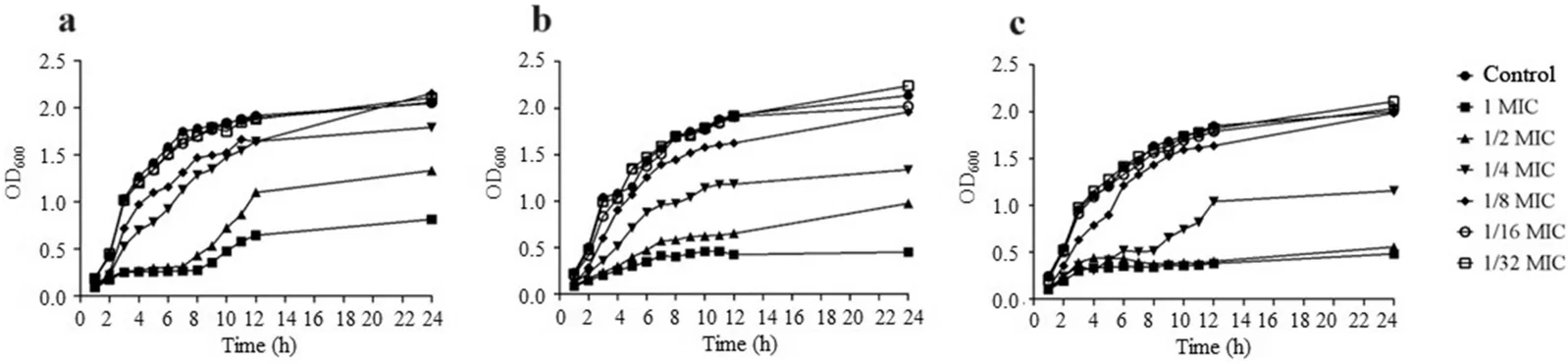
Growth curves of *S. aureus* at different sub-MICs of iclaprim. **a** *S. aureus* MW2; **b** *S. aureus* N315; **c** *S. aureus* ATCC 25923; control, no antibiotic

The MICs of iclaprim against the studied strains (MW2, N315, and ATCC25923) were 0.25 μg/mL. Figure 1 showed the influence of sub-MICs of iclaprim on the growth of *S. aureus*. Based on the growth curves, iclaprim at concentrations of 1/8*–*1/2 MICs significantly inhibited the growth of the three strains. However, in the presence of 1/16 and 1/32 MICs, the bacterial growth state was consistent with that of the control (no antibiotic). Therefore, both concentrations were used to study the effect of iclaprim on *S*. *aureus* virulence. This approach helped to rule out the effect of bacterial growth inhibition on virulence.

### Biofilm formation inhibition and eradication

**Fig. 2:**
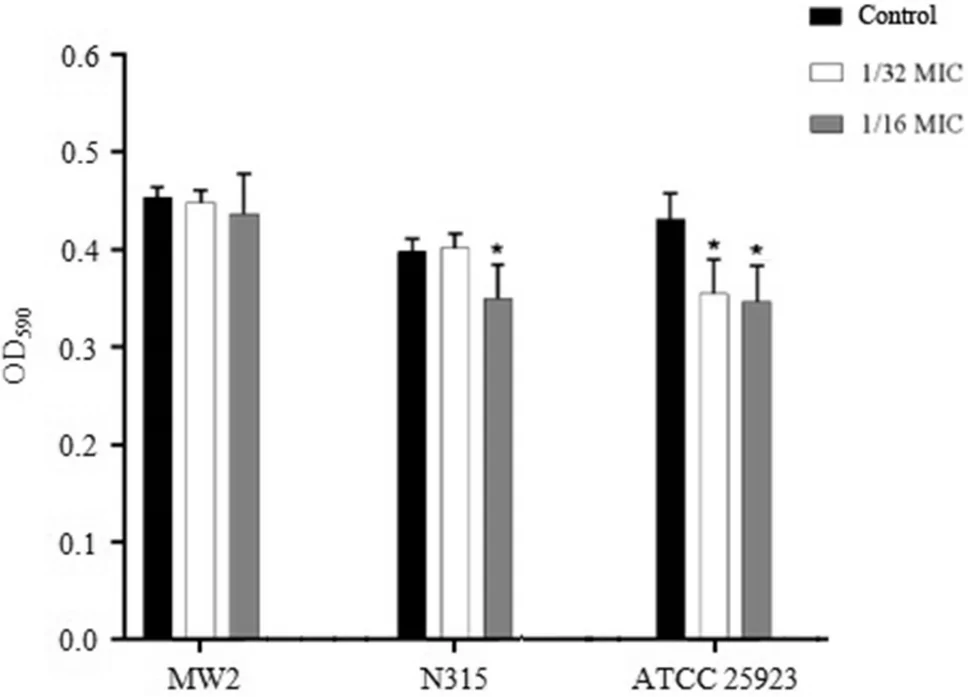
The effect of sub-MICs of iclaprim on the biofilm inhibition of *S. aureus* MW2, N315 and ATCC 25923. *P < 0.05

**Fig. 3:**
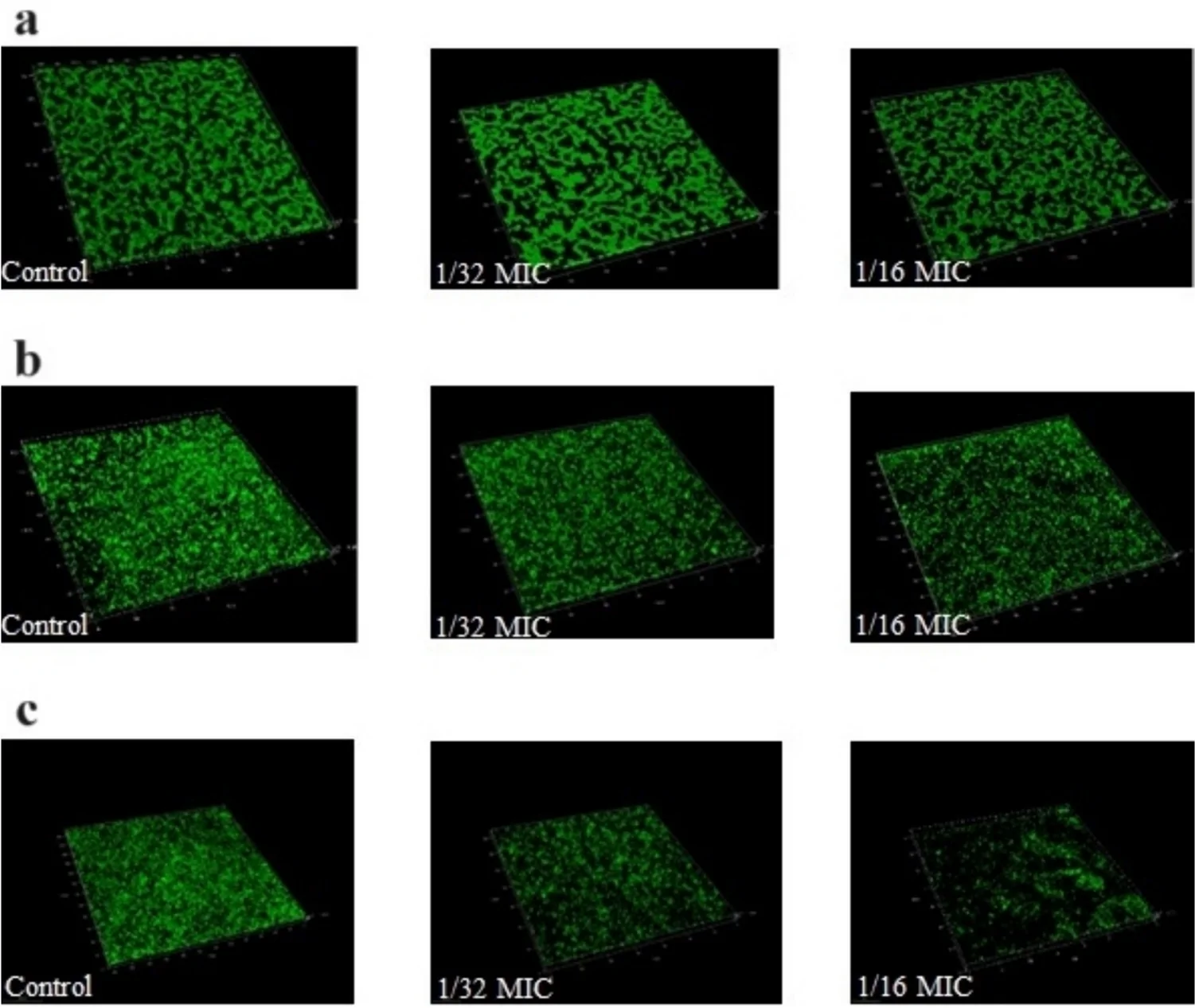
The role of sub-MICs of iclaprim on the biofilm inhibition of *S. aureus* under laser scanning confocal microscopy (LSCM) observation. **a** *S. aureus* MW2; **b** *S. aureus* N315; **c** *S. aureus* ATCC 25923

**Fig. 4:**
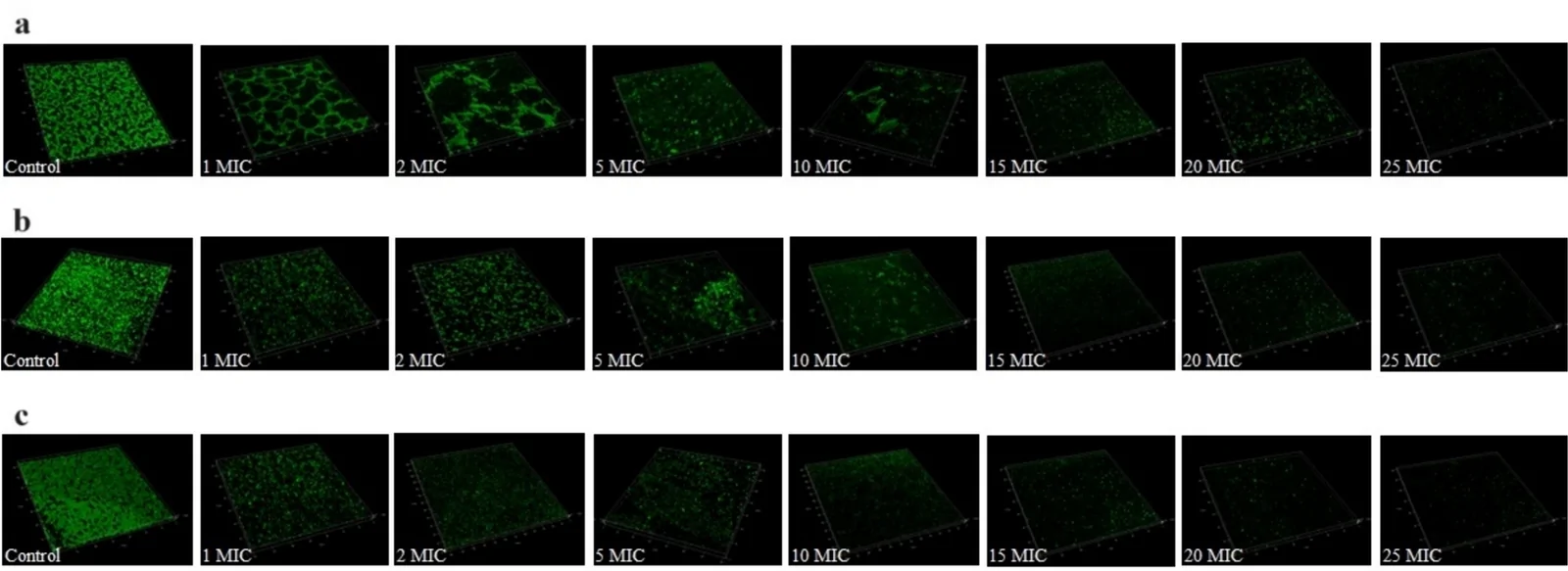
The eradication role of iclaprim on the mature biofilm of *S. aureus*. **a** *S. aureus* MW2; **b** *S. aureus* N315; **c** *S. aureus* ATCC 25923

The sub-MICs of iclaprim had a strain-dependent effect on the biofilm formation of *S. aureus*. Results from CV staining showed that the capacity for biofilm formation in strain N315 (1/16 MIC, *P* = 0.032) and ATCC 25923 (1/16 and 1/32 MICs, *P* = 0.03 and *P* = 0.028, respectively) was reduced by sub-MICs of iclaprim, while there was no significant effect on strain MW2 (Fig. 2). To further confirm the results of the CV staining, LSCM observation was performed. As shown in Fig. 3, the fluorescence area in the graphs of strain N315 (1/16 MIC) and ATCC 25923 (1/16 and 1/32 MICs) was less than that of the control group at sub-MICs of iclaprim (Fig. 3b and c). The green color range of strain MW2 did not show a marked difference at both concentrations detected (Fig. 3a). The observation results of the role of iclaprim on bacterial biofilm formation under LSCM were consistent with those of CV staining. Figure 4 showed the biofilm eradication of iclaprim: In the control, the mature biofilm formed on the culture dish wall exhibited a dense net-like film structure with a large fluorescence area, but when treated with 1 to 25 MICs of iclaprim, many cavities were formed in the biofilm of the tested strains, the net-like structure was destroyed, and the fluorescence area was greatly reduced. These phenomena indicate that iclaprim with concentrations of ≥ 1 MIC has the ability to depolymerize the mature biofilm of *S. aureus*.

### Effect of iclaprim on virulence

#### Effect on the activity of hemolysins and exoenzymes

As displayed in Table 1, iclaprim reduced the hemolytic activity of Hla in strains MW2 (*P* = 0.038) and ATCC 25923 (*P* = 0.013) at 1/16 MIC. It also decreased the activity of Hla in strain N315 at both sub-MICs studied (1/16 MIC, *P* = 0.007; 1/32 MIC, *P* = 0.012). However, iclaprim did not affect the Hld activity in all tested strains. The activity of coagulase in strain MW2 was inhibited by two-fold at 1/16 MIC of iclaprim but promoted by two-fold in *S. aureus* N315 at 1/16 and 1/32 MICs. Both sub-inhibitory concentrations of iclaprim had no effect on the activity of coagulase in strain ATCC 25923 (Table 1). Furthermore, Table 1 showed that iclaprim had no significant effect on the total activities of protease, nuclease, and lipase in all tested strains exposed to sub-MICs.

**Table 1:** Hemolytic and extracellular enzymatic activities and SpA production of *Staphylococcus aureus* impacted by sub-inhibitory concentrations of iclaprim

Toxin/enzyme/protein	Strain MW2			Strain N315			Strain ATCC 25923		
	1/16 MIC	1/32 MIC	Control	1/16 MIC	1/32 MIC	Control	1/16 MIC	1/32 MIC	Control
Hla activity^**a**^	94.22 ± 2.24* (*P* = 0.038)	100.04 ± 1.83	100.00	84.74 ± 3.13* (*P* = 0.007)	85.72 ± 2.14* (*P* = 0.012)	100.00	90.04 ± 1.94* (*P* = 0.043)	99.15 ± 1.87	100.00
Hld activity^**a**^	100.92 ± 2.10	98.41 ± 3.85	100.00	102.51 ± 1.94	101.11 ± 1.72	100.00	98.38 ± 2.1	101.92 ± 1.94	100.00
Coagulase titer^**b**^	1:2	1:4	1:4	1:64	1:64	1:32	1:2	1:2	1:2
Proteolytic activity^**a**^	97.34 ± 8.45	95.16 ± 5.49	100.00	102.16 ± 2.74	95.98 ± 6.14	100.00	99.81 ± 16.88	109.21 ± 14.09	100.00
Nuclease activity^**c**^	15 ± 0.2	15 ± 0.2	15 ± 0.3	17 ± 0.3	17 ± 0.2	17 ± 0.3	14 ± 0.6	15 ± 0.6	15 ± 0.3
Lipase activity^**a**^	124.16 ± 12.96	102.34 ± 7.82	100.00	132.16 ± 12.97	149.49 ± 22.30	100.00	134.98 ± 10.34	108.59 ± 3.14	100.00
SpA production^**d**^	29.57 ± 7.97 (P = 0.505)	32.13 ± 6.08 (P = 0.741)	33.37 ± 6.67	18.35 ± 4.72 (P = 0.362)	18.87 ± 2.25 (P = 0.219)	21.44 ± 2.15	28.72 ± 6.68 (P = 0.996)	28.88 ± 6.68 (P = 0.968)	28.62 ± 7.97
Hla production^**e**^	20.26 ± 1.29* (P = 0.011)	23.49 ± 0.40 (P = 0.490)	23.74 ± 0.41	17.36 ± 0.72* (P = 0.036)	17.64 ± 0.93* (P = 0.046)	21.44 ± 2.15	13.40 ± 0.41* (P = 0.046)	21.51 ± 2.67 (P = 0.92)	21.18 ± 4.72

^**a**^The supernatant of the drug-free treatment extract was used as a 100% hemolysis or lysis control. Values were the means ± standard deviation of findings of three repeat experiments. ^**b**^The maximum dilution of the sample coagulation was taken as the result. ^**c**^The data indicated the zone size (mm). ^**d**^The concentration unit of SpA was U/L. ^**e**^The concentration unit of α-hemolysin was ng/mL; *Hla* α-hemolysin *Hld* δ-hemolysin, *SpA* staphylococcal protein A, *P < 0.05; *Control* control group treated with 1% dimethylsulfoxide (DMSO)

#### Effect on the production of Hla and SpA

Table 1 shows that 1/16 and/or 1/32 MICs of iclaprim significantly inhibited the production of Hla. The results were consistent with those obtained from the activity detection. However, all tested strains were unaffected in terms of SpA protein levels under these conditions (Table 1).

#### Effect on the expression of virulence genes

**Fig. 5:**
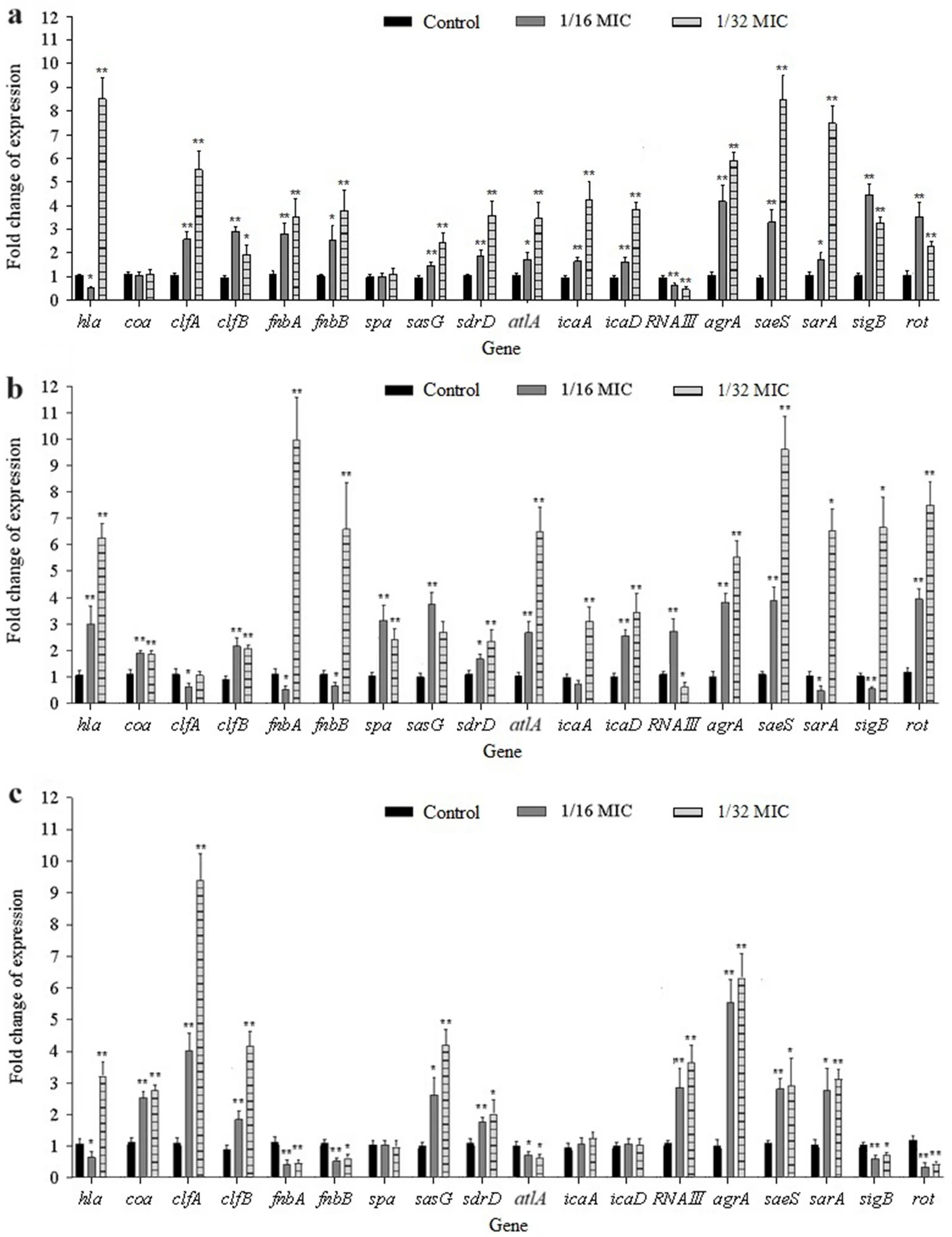
Effect of sub-MICs of iclaprim on transcription levels of virulence genes of *S. aureus* MW2 (**a**), N315 (**b**) and ATCC 25923 (**c**). The bars are the means of three independent experiments and the error bars represent the standard deviation. *P < 0.05; **P < 0.01

The expression of various virulence genes in the treated strains is shown in Fig. 5. In strain MW2, the expression of *clfA, clfB, fnbA, fnbB, sasG, sdrD, atlA, icaA, icaD, agrA, saeS, sarA, sigB,* and *rot* was significantly increased by 1/16–1/32 MICs of iclaprim, but the expression of RNAIII was suppressed by both concentrations (Fig. 5). Under these conditions, *hla* and *coa* expression levels were inhibited at 1/16 MIC, but *hla* expression was increased at 1/32 MIC (Fig. 5). No effect of sub-MICs of iclaprim was observed on the transcription of *spa* in this strain (Fig. 5). In strain N315, the transcription levels of *hla, spa, coa, clfB, sasG, sdrD, atlA, icaD, agrA, saeS,* and *rot* were observably stimulated by the graded concentrations of iclaprim. For genes *fnbA, fnbB, sarA,* and *sigB*, iclaprim led to a decrease in their mRNA levels at 1/16 MIC, but an increase at 1/32 MIC (Fig. 5). Increased RNAIII and reduced *clfA* mRNA levels were observed in strain N315 on exposure to iclaprim at 1/16 MIC (Fig. 5). However, decreased RNAIII and increased *icaA* expression levels were observed at 1/32 MIC (Fig. 5). In strain ATCC 25923, the expression of *coa, clfA, clfB, sasG, sdrD, RNAIII, agrA, saeS,* and *sarA* was significantly upregulated by 1/16–1/32 MICs of iclaprim, while the expression levels of *fnbA, fnbB, altA, sigB,* and *rot* were down-regulated at both concentrations (Fig. 5). For *hla*, iclaprim at 1/16 MIC resulted in a decrease in its mRNA level. In contrast, 1/32 MIC led to an increase. No effect was observed on the transcription of *spa, icaA,* and *icaD* in strain ATCC 25923 exposed to sub-MICs of iclaprim (Fig. 5). The differential folds and *P* values of the expression of various genes in studied strains were shown in Table S2.

### Iclaprim increases *G. mellonella* larvae survival

**Fig. 6:**
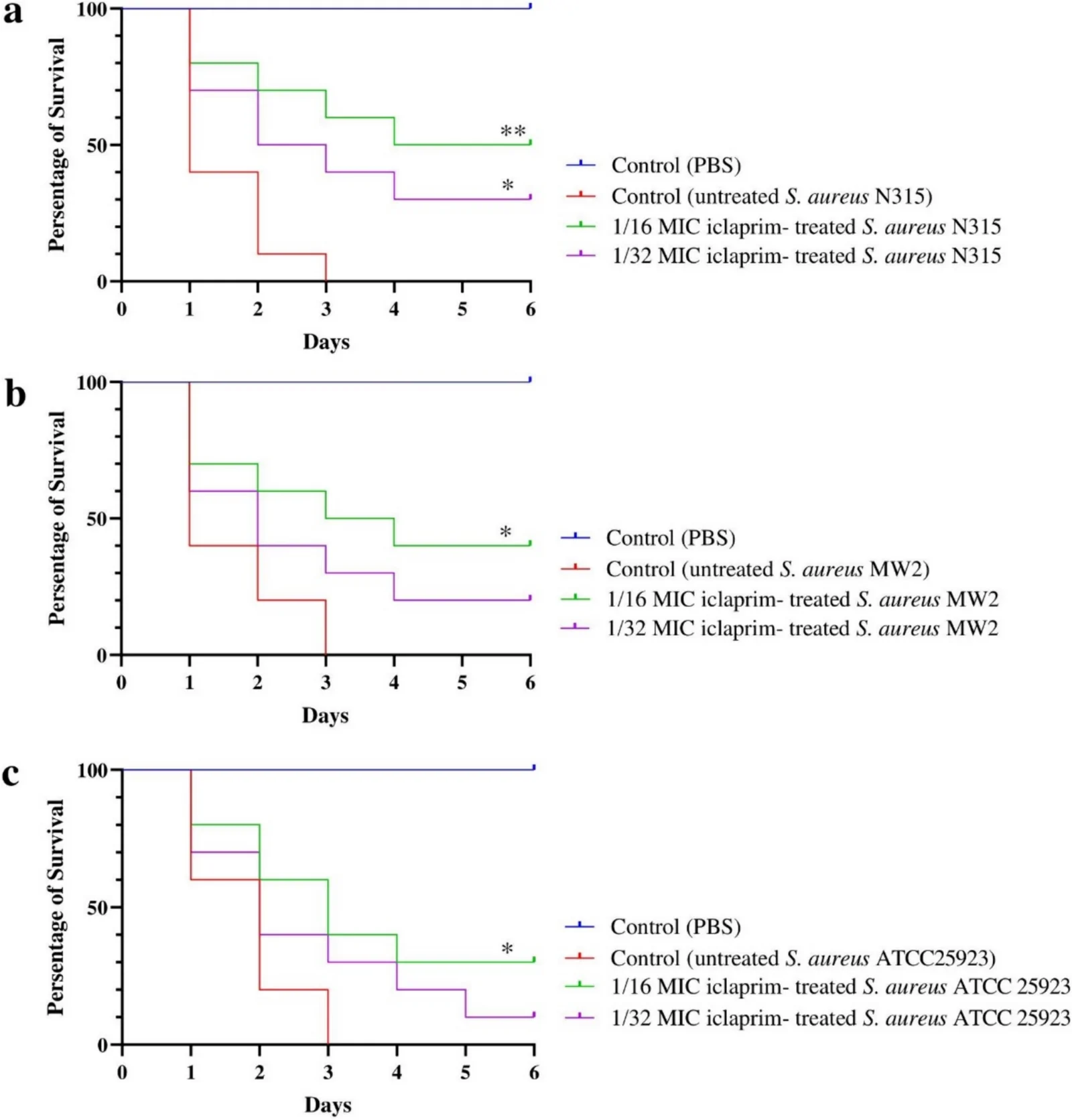
Survival curves for *Galleria mellonella* infected with sub-MICs of iclaprim treated *S. aureus*. **a** *S. aureus* MW2. **b*** S. aureus* N315. **c** *S. aureus* ATCC 25923. *G*. *mellonella* larvae received PBS and were assessed for 6 days post-infection. *n* = 10/group and the experiments were performed in duplicate. PBS, phosphate-buffered saline. *P < 0.05 and **P < 0.01 were considered with a striking difference, compared with untreated *S*. *aureus*-challenged larvae

As shown in Fig. 6, PBS (negative control) did not induce any toxicity on larvae; however, the larvae in the positive control groups all died in the first 3 days. In contrast, iclaprim greatly enhanced the survival rates of *G*. *mellonella* larval, as 40% (*P* = 0.0149), 50% (*P* = 0.0023), and 40% (*P* = 0.0281) remained alive on day 6, respectively, for 1/16 MIC of iclaprim treated strains MW2, N315, and ATCC25923. For the larvae infected by 1/32 MIC iclaprim treated strains, the 6-day survival rates were 10–30% (*P* < 0.05 (0.0246) only for strain N315, the survival rate was 30%).

## Discussion

The impact of antibacterial agents on microorganisms depends on their concentrations. Although high doses of drugs kill bacteria, doses below therapeutic levels can play a subtler role in bacterial physiology (Li et al. 2020). Sub-inhibitory concentrations of antibiotics, such as β-lactams, aminoglycosides, macrolides, fluoroquinolones, linezolid, and clindamycin, can dramatically regulate the production of virulence factors of bacteria (Chen et al. 2021; Hodille et al. 2017; Liu et al. 2021). This phenomenon can partially explain why certain agents have demonstrated decreased clinical effect in cases of human infection with some bacteria, while drugs that weaken the virulence of bacteria are significantly superior (Stevens et al. 2014). Here, we comprehensively explored the influence of sub-inhibitory concentrations of iclaprim on the virulence traits of *S. aureus*. Better understanding the ability of sub-inhibitory-dose iclaprim to serve as a modulating compound is conducive to optimizing the therapeutic strategy of this drug.

The antimicrobial mechanism of iclaprim is similar to that of trimethoprim, a first-generation DHFR inhibitor. However, iclaprim has a significantly greater binding ability to bacterial DHFR and the enzyme with F98Y mutation compared to trimethoprim (Oefner et al. 2009). Therefore, iclaprim could have wider clinical applications, which is the reason we chose this drug for evaluation.

Hla and Hld, known as cytolysins, are important virulence factors produced by *S*. *aureus*, which can form pore channels across the cell membrane and lysis targeted cells (Harmsen et al. 2003; Otto 2014; Verdon et al. 2009). Previous studies showed that multiple antibiotics at sub-inhibitory levels, such as ciprofloxacin, macrolides, clindamycin, and oxazolidinones, could inhibit the production of Hla (Chen et al. 2021). In this study, sub-MICs of iclaprim reduced the Hla activity and the toxin protein expression in all *S*. *aureus* tested in a strain-dependent manner, and the results were also consistent with those of Bryant’s study (Bryant et al. 2019). However, genetically, the transcription levels of *hla* did not align with the activity and production of the toxin. For instance, in strain N315, the mRNA levels of *hla* increased with iclaprim at sub-MIICs. However, the activity and production of Hla decreased. Meanwhile, in strains MW2 and ATCC 25923, the expression of *hla* was stimulated at 1/32 MIC, but the activity and production of the toxin remained unaffected (Table 1 and Fig. 5). It should be noted that, regardless of the expression level of *RNAIII* (the Hld-encoding gene) under sub-MICs of iclaprim conditions, the activity of Hld was not different in the studied strains. Sub-MICs of iclaprim could also control the activity of coagulase in a strain-dependent manner, namely decreased in strain MW2, increased in strain N315, and no change in strain ATCC 25923. Similar to the situation with *hla* expression, the *coa* expression level was also inconsistent with coagulase activity at sub-MICs of iclaprim, meaning that *coa* expression was increased but the enzyme activity was not affected in strain ATCC25923, while *coa* expression was unchanged but the enzyme activity was decreased in the MW2 strain. It is well known that due to the complex regulated mechanisms (such as disturbance of ribosome function, transcriptional regulation, post-transcriptional/translational modifications, and interaction between regulatory networks) of gene transcription and translation, changes in the mRNA levels do not always lead to alterations in protein production (Hodille et al. 2017; Atshan et al. 2021). This situation is formed by the perplexing balance of these responses, which are closely associated with the genetic backgrounds of bacterial strains (Hodille et al. 2017). Therefore, we believe that the discordance between transcription level and protein synthesis or/and functional activity for Hla and coagulase might be caused by the different genetic background of the strains or the mutation or deletion of some significant regulators (Blevins et al. 2002; Hodille et al. 2017).

It is well known that the formation of bacterial biofilm plays a greatly important role in resistance to antibiotic and disinfectant, hostile environment, and host immune system; therefore, it is related to chronic or recurrent infections (Schilcher and Horswill 2020). It has become a consensus that antibiotics at sub-MICs influence bacterial biofilm formation by interfering with certain stages of development (Chen et al. 2021). Previous studies showed that sub-inhibitory dose of trimethoprim could prevent the biofilm development of *Acinetobacter baumannii* (Moon et al. 2017). Therefore, we hypothesized that low concentrations of iclaprim, in the same class as trimethoprim, could also repress *S*. *aureus* biofilm. In this study, two out of three tested strains showed reduced biofilm production at sub-MICs of iclaprim, verifying that drug treatments at tiny dose regulated the community structure and heterogeneity of *S*. *aureus* biofilm. Previous study reported that trimethoprim eradicates the biofilms of uropathogenic *Escherichia coli* at the concentrations of 512–1024 μg/mL (Rafaque et al. 2020). Our results showed that iclaprim had a good function in removing the mature biofilm of *S*. *aureus*, and the destructive effect could be produced at 0.25 μg/mL (1 MIC), which is far less than those of trimethoprim to clear *E*. *coli* biofilm.

Inhibition of biofilm formation is associated with reduced expression of bacterial surface proteins, such as ClfA, ClfB, FnbA, FnbB, SpA, SasG, and SdrD (Peng et al. 2022; Schilcher and Horswill 2020; Valle et al. 2019). These proteins contain a conserved cell wall-anchoring motif of Leu-Pro-Xxx-Thr-Gly (LPXTG, X stands for any amino acid) at the C-terminus and can mediate initial surface attachment (the first phase) in the progress of staphylococcal biofilm formation (Blevins et al. 2002; Valle et al. 2019). During biofilm maturation process, a major biofilm matrix component is polysaccharide intercellular adhesin (PIA), whose synthesis is mediated by the *icaADBC* operon. The *icaA* (encoding transmembrane glycosyltransferase) and *icaD*, the first two genes in this gene cluster, carry out key role in the exopolysaccharide production (Blevins et al. 2002; Speziale et al. 2014). The impacts of the sub-MICs of drugs on the expression of the adhesive molecules may change depending on the *S*. *aureus* strain tested (Chen et al. 2021). For example, Yang et al. (2017) found that kanamycin, enrofloxacin, lincomycin, clarithromycin, and colistin sulfate can repress the development of biofilm and the expression of the biofilm-related genes of *fnbA*, *rbf*, *eno*, *lrgA*, *cidA*, and *sarA* in *S*. *aureus* clinical strain Hb0206. Another study conducted by Jo and Ahn (2016) found that 1/2 MIC of levofloxacin dramatically increased the expression of adhesion-related genes *fnbA*, *fnbB*, *clfA*, *clfB*, and *icaD* in *S*. *aureus* strain CCARM 308, but reduced that of genes *icaA* and *icaD* in strain KACC 10778. In the present study, the strain-dependent regulative effects of sub-MICs of iclaprim on the adhesion genes expression was also observed. Although increased expression of *clfB*, *sasG*, *sdrD*, *icaA*, and *icaD* were observed in strains N315 and ATCC 25923, the enhancement role in biofilm formation was not found, especially for strain MW2, which almost up-regulated all the factors studied. This contradicted previous statement that the up-regulated levels of adhesion-related genes were directly associated with the reinforced capacity of biofilm formation (Hoiby et al. 2010). However, the reduction in the expression of a few factors, such as *fnbA* and *fnbB*, was displayed to be consistent with the decreased biofilm formation. The reason for this might be that sub-MIC of iclaprim affected the expression of genes at the transcriptional level, but did not affect their expression at protein level, which is the functional form that really plays a role. For example, in strains, N315 increased expression of *spa* did not result in the increase of SpA protein (Table 1 and Fig. 5).

Protease, nuclease (degrading extracellular DNA, eDNA), and lipase (promoting the release of eDNA) are key virulence factors in *S*. *aureus*, which are associated with the biofilm formation and the immune evasion of bacteria (Nguyen et al. 2018; Patel and Rawat 2023). In this study, we did not observe the alteration of the total activity of the three kinds of enzymes in the studied strains after treatment by sub-MICs iclaprim (Table 1). Accordingly, we believe that the inhibition of biofilm formation is not correlated to the extracellular enzymes-mediated instability of the biofilm. Previous studies suggested that the activity of the main autolysin AtlA in *S*. *aureus* can facilitate the surface attachment and the release of eDNA during the formation of biofilm (Götz et al. 2014). However, the increased mRNA levels of *atlA* did not promote the formation of biofilm in partial strains tested.

The sub-inhibitory influences of iclaprim are not just restricted to biofilm formation and the expression of adhesion-related factors. The drug has a broader effect leading to the changed expression of important two-component system (TCS)-associated determinants and global regulators, such as Agr system, Sae system, Sar system, SigB, RNAIII, and Rot, and ultimately affecting virulence (Schilcher and Horswill 2020). AgrA, acting as response regulator of Agr system, directly stimulates the expression of RNAII transcriptional unit of *agr* locus, resulting in increased AIP production and a positive feedback circuit. Besides the direct role, AgrA also induces the expression of the primary effector RNAIII of *agr* operon, which can control the biofilm formation and disassembly by upregulating the genes encoding toxins and exoenzymes and repressing the genes encoding surface-associated proteins (Jenul and Horswill 2019). Sae locus, consisting of sensor histidine kinase SaeS and response regulator SaeR, is also crucial for biofilm maturation and pathogenesis by driving the expression of many exoproteins and adhesins, such as Hla, nuclease, and surface proteins FnbA/B (Jenul and Horswill 2019; Schilcher and Horswill 2020). In addition, SaeRS also directly regulates the exodus event (early biofilm dispersal) of biofilm. Global regulator SarA stimulates the expression of some virulence factors, *agr* system, *hla*, *fnbA*, and *fnbB*, but represses the production of SpA. The most critical role of SarA in biofilm formation is promoting the development of biofilm by the inhibition of protease production (Jenul and Horswill 2019). In addition, SarA can work collaboratively with Sae system to reduce the production of proteases and drive the formation of biofilm in *S*. *aureus* (Schilcher and Horswill 2020). Rot, belonging to SarA family, also showed a significant increase in biofilm formation by promoting the expression of surface proteins and repressing the production of toxins and exoenzymes. SigB, a transcription factor for stress responses, can regulate the expression of more than 250 genes; among them, genes for adhesins (such as FnbA and ClfA, mediating the primary phases of biofilm formation) are stimulated and genes for exoenzymes (such as proteases and nuclease, for biofilm dispersal) are repressed (Schilcher and Horswill 2020). Additionally, SigB can also repress the expression of RNAIII, Sae system and AIP production depending on the strain genetic background or environmental conditions (Geiger et al. 2008; Schilcher and Horswill 2020; Speziale et al. 2014).

In the current study, the situations of the increased expression of *agrA*, *saeS*, and *RNAIII* and the reduced expression of *sigB* and *rot* were consistent with the reduction in the ability of biofilm formation in strain ATCC 25923 under the sub-MICs of iclaprim; however, the increased expression of *sarA* did not activate the biofilm formation. For strain N315, except *sarA* and *rot*, the situations was similar as that of strain ATCC 25923 under 1/16 MIC of iclaprim. In strain MW2, regardless of the increased or decreased mRNA levels, these regulatory genes did not affect the biofilm formation ability.

Although we analyzed the aforementioned multiple factors that promote or reduce the formation of biofilms (such as the expression of adhesion related genes and regulatory genes, and the production of extracellular proteins), the underlying reason of iclaprim resulting in heterogeneity in the virulence factor expression and the biofilm formation ability of the strains in this study is not clear, and likely to be linked to their genetic background, or the signals in different physiological pathways or stress conditions induced by this drug modulating multiple response regulators (Atshan, et al. 2021).

Finally, the impacts of sub-MICs of iclaprim on virulence of *S*. *aureus* were estimated again by the *G*. *mellonella* infection model, which has been widely applied to evaluate the virulence of bacteria (Ménard et al. 2021; Serrano et al. 2023). Our findings suggest that 1/16 MIC of iclaprim could prominently lengthen the survival time of the infected *G*. *mellonella* larvae; however, a significant effect of lower concentration (such as 1/32 MIC) of drug on survival showed strain-dependent. Combined with the results of phenotypic experiments *in vitro*, we speculated that this situation may be mainly related to the inhibition of Hla production mediated by iclaprim (iclaprim at 1/32 MIC only reduced the production of Hla in N315) (Bryant et al. 2019).

In summary, the extensive use of antibacterial agents has led to the development and prevalence of antibiotic-resistant *S. aureus*. Anti-virulence therapies that repress bacterial virulence, such as toxin, exoenzyme, regulatory factor production, and biofilm formation, offer potential therapeutic regimens for appropriately treating infections caused by antibiotic-resistant *S. aureus*. Our study provides insight into the diverse characteristics of virulence in representative strains of *S. aureus*, such as MW2, N315, and ATCC 25923, when exposed to sub-MICs of iclaprim. Based on our results, we suggest that iclaprim effectively inhibits Hla production and biofilm formation at sub-MICs in a strain-dependent manner and is an excellent depolymerizing agent of mature biofilm at concentrations of ≥ 1 MIC. Additionally, our study documents the differing effects of sub-MIC iclaprim on the expression of adhesion and regulation-related genes, as well as the activity of coagulase, and the impact appears to be also dependent on the strain. The increased survival rate of challenged larvae further verifies the good performance of sub-lethal iclaprim, which offers significant sight into fighting *S*. *aureus*. Overall, our findings indicate that sub-MIC iclaprim may alter the pathogenesis of *S. aureus*, thereby limiting the progression of infections. This information is significant in establishing valuable therapeutic strategies to improve outcomes for patients with *S. aureus* diseases.

## Supplementary Information

Below is the link to the electronic supplementary material.

Supplementary file1 (PDF 308 KB)

## Data Availability

Data for this study are available upon request from the corresponding author.
