# Supplementary material for: Anti-virulence potential of iclaprim, a novel folic acid synthesis inhibitor, against Staphylococcus aureus
Source: Appl Microbiol Biotechnol. 2024 Aug 5;108:432. doi: 10.1007/s00253-024-13268-2 (PMC11300511; doi:10.1007/s00253-024-13268-2)
Supplement: Supplementary file 1 [file 253_2024_13268_MOESM1_ESM.pdf]

Anti-virulence potential of iclaprim, a novel folic acid synthesis inhibitor, against *Staphylococcus aureus*

Applied Microbiology and Biotechnology

Lingyun Hao[1,†], Jingwen Zhou[2,†], Han yang[1], Chunyan He[1], Wen Shu[1], Haoyue Song[1], Qingzhong Liu[2,*]

[1] Department of Clinical Laboratory, Shanghai General Hospital, Shanghai Jiaotong University School of Medicine; Shanghai, China

[2] Department of Clinical Laboratory, Shanghai Municipal Hospital of Traditional Chinese Medicine, Shanghai University of Traditional Chinese Medicine; Shanghai 200071, China.

Lingyun Hao: haolingyun_sjtu@163.com

Jingwen Zhou: zhoujingwenstudy@163.com

Han yang: yanghansjtu@163.com

Chunyan He: hechunyan_hcy@163.com

Wen Shu: shuwen654321@163.com

Haoyue Song: songhaoyuex@sina.com

Qingzhong Liu: jiaodamedicine@foxmail.com

[†]Lingyun Hao and Jingwen Zhou contributed equally to this work.

*Corresponding author's mailing address: Department of Clinical Laboratory, Shanghai Municipal Hospital of Traditional Chinese Medicine, Shanghai University of Traditional Chinese Medicine, 274 Zhijiang Middle Rd., Shanghai, People's Republic of China; Zip Code: 200071. E-mail: jiaodamedicine@foxmail.com

Running title: Anti-virulence capacity of iclaprim on *S. aureus*

Supplementary Table 1 Virulence genes and primers used for qRT-PCR

Gene	Sequence of primer（5'-3'）	References
hla-F	AATGAATCCTGTCGCTAATGCCGC	(Xiong et al. 2006)
hla-R	CTGAAGGCCAGGCTAAACCACTTT	
coa-F	AGGTCTTGAAGGTAGCTCAT	(Blickwede et al. 2005)
coa-R	GTTGTATTCACGGATACCTGTA	
clfA-F	TGCTGCACCTAAAACAGACG	(Kim et al. 2017)
clfA-R	TGTGTCGTTTCCTGTTGTGC	
clfB-F	TTGCCGCCATAAAAGTATTA	(Blickwede et al. 2005)
clfB-R	TCACCACAAACAATTTCCAA	
fnbA-F	ACAAGTTGAAGTGGCACAGCC	(Blickwede et al. 2005)
fnbA-R	CCGCTACATCTGCTGATCTTGTC	
fnbB-F	CGTTATTTGTAGTTGTTTGTGTT	(Blickwede et al. 2005)
fnbB-R	TGGAATGGGACAAGAAAAAGAA	
spa-F	GCGCAACACGATGAAGCTCAACAA	(Blickwede et al. 2005)
spa-R	ACGTTAGCACTTTGGCTTGGATCA	
sasG-F	GGTTTTCAGGTCCTTTTGGAT	(Blickwede et al. 2005)
sasG-R	CTGGTGAAGAGCGAGTGAAA	
sdrD-F	AGTACACAGTGGGAACAGCATC	(Blickwede et al. 2005)
sdrD-R	TCTGCAGCCTTTGCTTCTTGGTTC	
atlA-F	TGTCGAAGTATTTGCCGACTTCGC	(Bose et al. 2012)
atlA-R	TGGAATCCTGCACATCCAGGAAC	

icaA-F	AACAGAGGTAAAGCCAACGCACTC	(Wang et al. 2010)
icaA-R	CGATAGTATCTGCATCCAAGCAC	
icaD-F	ATGGTCAAGCCCAGACAGAG	(Wang et al. 2010)
icaD-R	AGTATTTTCAATGTTTAAAGCAA	
RNAIII-F	TAATTAAGGAAGGAGTGATTTCAATG	(Boisset et al. 2007)
RNAIII-R	TTTTTAGTGAATTTGTTCACTGTGTC	
agrA-F	TGATAATCCTTATGAGGTGCTT	(Boisset et al. 2007)
agrA-R	CACTGTGACTCGTAACGAAAA	
saeS-F	ATCCGAACAACAAGAAAAAACAG	(Shang et al. 2009)
saeS-R	TGATTATACCATCACGTAGTCCTTCA	
sarA-F	CCTCGCAACTGATAATCCTTATG	(Andrey et al. 2010)
sarA-R	ACGAATTTCACTGCCTAATTTGA	
sigB-F	TCAGCGGTTAGTTCATCGCTCACT	(Tran and Bonilla 2021)
sigB-R	GTCCTTTGAACGGAAGTTTGAAGCC	
rot-F	AAGAGCGTCCTGTTGACGAT	(Jefferson et al. 2004)
rot-R	TTTGCATTGCTGTTGCTCTA	
16S rRNA-F	CGTGCTACAATGGACAATACAAA	(Zhou et al. 2022)
16S rRNA-R	ATCTACGATTACTAGCGATTCCA	

qRT-PCR, quantitative real-time polymerase chain reaction

Reference

Andrey DO, Renzoni A, Monod A, Lew DP, Cheung AL, Kelley WL (2010) Control of the *Staphylococcus aureus* toxic shock tst promoter by the global regulator SarA. J Bacteriol 192(22):6077-85. https://doi.org/10.1128/JB.00146-10

Blickwede M, Wolz C, Valentin-Weigand P, Schwarz S (2005) Influence of clindamycin on the stability of coa and fnbB transcripts and adherence properties of *Staphylococcus aureus* Newman. FEMS Microbiology Letters 252(1):73-78. https://doi.org/10.1016/j.femsle.2005.08.022

Boisset S, Geissmann T, Huntzinger E, Fechter P, Bendridi N, Possedko M, Chevalier C, Helfer AC, Benito Y, Jacquier A, Gaspin C, Vandenesch F, Romby P (2007) *Staphylococcus aureus* RNAIII coordinately represses the synthesis of virulence factors and the transcription regulator Rot by an antisense mechanism. Genes Dev 21(11):1353-66. https://doi.org/10.1101/gad.423507

Bose JL, Lehman MK, Fey PD, Bayles KW (2012) Contribution of the *Staphylococcus aureus* Atl AM and GL murein hydrolase activities in cell division, autolysis, and biofilm formation. PLoS One 7(7):e42244. https://doi.org/10.1371/journal.pone.0042244

Jefferson KK, Pier DB, Goldmann DA, Pier GB (2004) The teicoplanin-associated locus regulator (TcaR) and the intercellular adhesin locus regulator (IcaR) are transcriptional inhibitors of the ica locus in *Staphylococcus aureus*. J Bacteriol 186(8):2449-56. https://doi.org/10.1128/JB.186.8.2449-2456.2004

Kim MK, Zhao A, Wang A, Brown ZZ, Muir TW, Stone HA, Bassler BL (2017)

Surface-attached molecules control *Staphylococcus aureus* quorum sensing and biofilm development. Nat Microbiol 2:17080. https://doi.org/10.1038/nmicrobiol.2017.80

Shang F, Xue T, Sun H, Xing L, Zhang S, Yang Z, Zhang L, Sun B (2009) The *Staphylococcus aureus* GGDEF domain-containing protein, GdpS, influences protein A gene expression in a cyclic diguanylic acid-independent manner. Infect Immun 77(7):2849-56. https://doi.org/10.1128/IAI.01405-08

Tran HT, Bonilla CY (2021) SigB-regulated antioxidant functions in gram-positive bacteria. World J Microbiol Biotechnol 37(3):38. https://doi.org/10.1007/s11274-021-03004-7

Wang Q, Sun FJ, Liu Y, Xiong LR, Xie LL, Xia PY (2010) Enhancement of biofilm formation by subinhibitory concentrations of macrolides in icaADBC-positive and -negative clinical isolates of Staphylococcus epidermidis. Antimicrob Agents Chemother 54(6):2707-11. https://doi.org/10.1128/AAC.01565-09

Xiong YQ, Willard J, Yeaman MR, Cheung AL, Bayer AS (2006) Regulation of *Staphylococcus aureus* alpha-toxin gene (hla) expression by agr, sarA, and sae in vitro and in experimental infective endocarditis. The Journal of Infectious Diseases 194(9):1267-1275. https://doi.org/10.1128/10.1086/508210

Zhou J, Zhao H, Yang H, He C, Shu W, Cui Z, Liu Q (2022) Insights Into the Impact of Small RNA SprC on the Metabolism and Virulence of *Staphylococcus aureus*. Front Cell Infect Microbiol 12:746746. https://doi.org/10.3389/fcimb.2022.746746

Supplementary Table 2. The differential folds and P values of the expression of various genes influenced by grade concentrations of iclaprim in strains of MW2, N315 and ATCC 25923

Gene		MW2+no antibiotic		MW2+1/16 MIC iclaprim				MW2+1/32 MIC iclaprim			
		**Mean**	**Std Deviation**	**Mean**	**Std Deviation**	**Fold Change**	***p* value**	**Mean**	**Std Deviation**	**Fold Change**	***p* value**
Exoenzyme genes	*hla*	1.000	0.17	-1.79	0.07	0.56	0.001	8.56	1.39	8.56	0.001
	coa	1.000	0.11	0.91	0.18	0.91	0.43	1.2	0.21	1.2	0.56
Adhesion genes	*clfA*	1.000	0.2	2.58	0.39	2.58	0.007	5.53	1.18	5.53	0.004
	clfB	1.000	0.15	2.94	0.24	2.94	0.001	1.94	0.48	1.94	0.016
	fnbA	1.000	0.22	2.82	0.57	2.82	0.003	3.53	1.12	3.53	0.006
	fnbB	1.000	0.09	2.54	0.71	2.54	0.014	3.82	1.21	3.82	0.004
	spa	1.000	0.11	1.04	0.08	1.04	0.37	1.16	0.24	1.16	0.28
	sasG	1.000	0.21	1.49	0.08	1.49	0.03	2.48	0.36	2.48	0.003
	sdrD	1.000	0.15	1.86	0.22	1.86	0.005	3.59	0.88	3.59	0.002
	altA	1.000	0.22	1.72	0.31	1.72	0.024	3.48	0.86	3.48	0.003
	icaA	1.000	0.08	1.79	0.18	1.79	0.01	6.62	0.93	6.62	0.011
	icaD	1.000	0.07	1.63	0.2	1.63	0.006	3.86	0.41	3.86	0.001
Regulator genes	*RNAIII*	1.000	0.21	-1.52	0.05	0.66	0.021	-2.06	0.06	0.49	0.003
	agrA	1.000	0.2	4.19	0.72	4.19	0.002	5.93	0.37	5.93	<0.001
	saeS	1.000	0.22	3.33	0.46	3.33	0.001	8.48	1.43	8.48	<0.001
	sarA	1.000	0.14	1.74	0.27	1.74	0.015	7.51	0.96	7.51	<0.001
	sigB	1.000	0.13	4.47	0.37	4.47	<0.001	3.3	0.19	3.3	<0.001
	rot	1.000	0.22	3.54	0.72	3.54	0.003	2.3	0.21	2.3	0.02

Gene		N315+no antibiotic		N315+1/16 MIC iclaprim				N315+1/32 MIC iclaprim			
		**Mean**	**Std Deviation**	**Mean**	**Std Deviation**	**Fold Change**	***p* value**	**Mean**	**Std Deviation**	**Fold Change**	***p* value**
Exoenzyme genes	*hla*	1.000	0.23	3	0.83	3	0.009	6.62	0.71	6.62	0.01
	coa	1.000	0.2	1.89	0.18	1.89	0.002	1.86	0.21	1.86	0.004
Adhesion genes	*clfA*	1.000	0.31	-1.52	0.11	0.66	0.02	0.9	0.13	0.9	0.314
	clfB	1.000	0.19	2.17	0.33	2.17	0.003	2.07	0.17	2.07	0.01
	fnbA	1.000	0.22	-1.58	0.16	0.63	0.011	6.26	1.81	6.26	0.002
	fnbB	1.000	0.16	-1.52	0.14	0.66	0.017	6.26	1.92	6.26	0.006
	spa	1.000	0.19	3.15	0.67	3.15	0.003	2.41	0.49	2.41	0.005
	sasG	1.000	0.15	3.76	0.52	3.76	<0.001	2.7	0.47	2.7	0.002
	sdrD	1.000	0.22	1.67	0.21	1.67	0.012	2.35	0.52	2.35	0.008
	altA	1.000	0.21	2.67	0.51	2.67	0.003	6.51	1.05	6.51	<0.001
	icaA	1.000	0.22	1.79	0.14	1.79	0.157	6.26	0.82	6.26	0.004
	icaD	1.000	0.17	2.57	0.22	2.57	<0.001	3.46	0.84	3.46	0.004
Regulator genes	*RNAIII*	1.000	0.08	2.73	0.5	2.73	0.004	-1.64	0.22	0.61	0.012
	agrA	1.000	0.15	3.83	0.44	3.83	0.01	5.54	0.75	5.54	<0.001
	saeS	1.000	0.09	3.89	0.61	3.89	0.001	9.62	1.37	9.62	<0.001
	sarA	1.000	0.13	2.04	0.22	2.04	0.014	6.28	0.98	6.28	0.012
	sigB	1.000	0.1	-1.81	0.13	0.55	0.002	6.34	1.29	6.34	0.021
	rot	1.000	0.11	3.91	0.46	3.91	0.008	7.48	1.04	7.48	<0.001

Gene		ATCC25923+no antibiotic		ATCC25923+1/16 MIC iclaprim				ATCC25923+1/32 MIC iclaprim			
		**Mean**	**Std Deviation**	**Mean**	**Std Deviation**	**Fold Change**	***p* value**	**Mean**	**Std Deviation**	**Fold Change**	***p* value**
Exoenzyme genes	*hla*	1.000	0.2	-1.52	0.18	0.66	0.04	3.23	0.51	3.23	0.01
	coa	1.000	0.18	2.53	0.27	2.53	0.001	2.78	0.22	2.78	0.009
Adhesion genes	*clfA*	1.000	0.31	4.04	0.63	4.04	0.001	9.42	1.16	9.42	0.001
	clfB	1.000	0.21	1.86	0.28	1.86	0.04	4.18	0.51	4.18	0.001
	fnbA	1.000	0.2	-2.22	0.11	0.45	0.004	-2.03	0.1	0.49	0.005
	fnbB	1.000	0.24	1.85	0.09	1.85	0.003	1.56	0.11	1.56	0.008
	spa	1.000	0.2	1.1	0.21	1.1	0.623	0.92	0.2	0.92	0.144
	sasG	1.000	0.11	2.62	0.61	2.62	0.03	4.23	0.54	4.23	0.001
	sdrD	1.000	0.14	1.76	0.13	1.76	0.005	2.04	0.48	2.04	0.023
	altA	1.000	0.21	-1.37	0.08	0.73	0.043	-1.56	0.14	0.64	0.021
	icaA	1.000	0.18	1.11	0.2	1.11	0.241	1.22	0.18	1.22	0.082
	icaD	1.000	0.15	1.13	0.19	1.13	0.133	1.06	0.21	1.06	0.18
Regulator genes	*RNAIII*	1.000	0.15	2.86	0.77	2.86	0.008	3.65	0.52	3.65	＜0.001
	agrA	1.000	0.24	5.51	0.82	5.51	0.001	6.32	0.84	6.32	＜0.001
	saeS	1.000	0.2	2.92	0.31	2.92	0.003	2.91	0.94	2.91	0.21
	sarA	1.000	0.11	2.77	0.82	2.77	0.013	3.13	0.32	3.13	＜0.001
	sigB	1.000	0.14	-1.62	0.17	0.62	0.008	-1.35	0.16	0.74	0.014
	rot	1.000	0.17	-2.94	0.18	0.34	0.03	-2.17	0.18	0.46	0.02

Figure S1 Calibration Curve-Hla and SPA

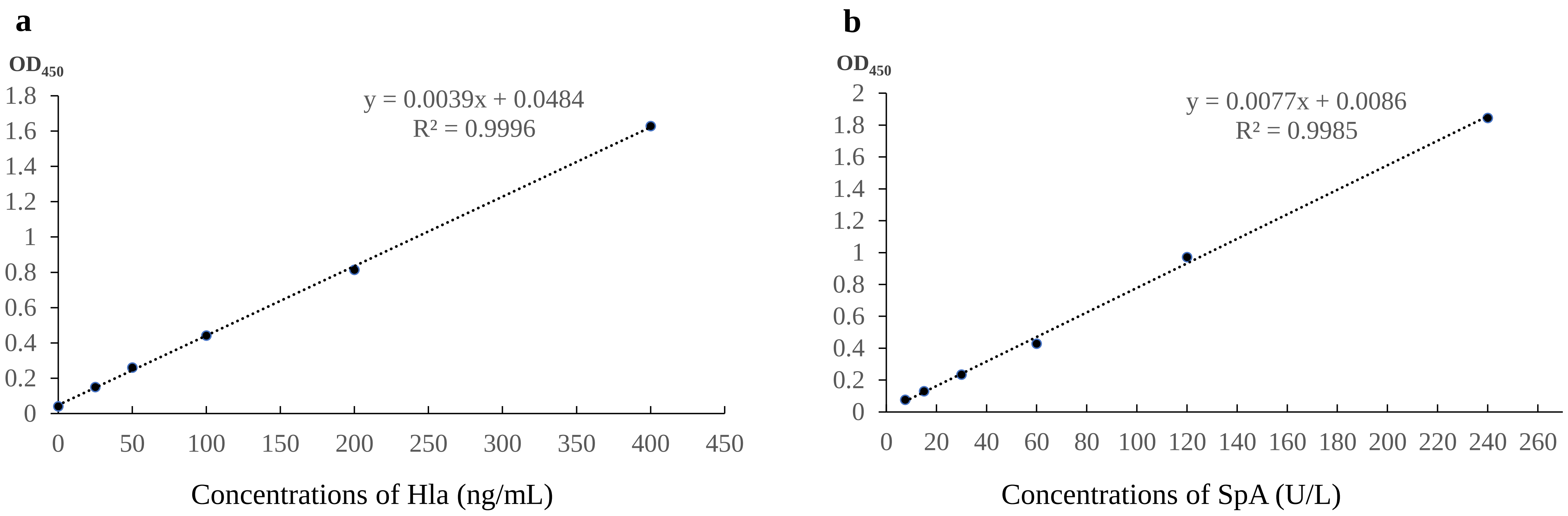

a Hla; b SpA. Hla, α-hemolysin; SpA, staphylococcal protein A
